# Cord blood cardiac markers and tissue doppler echocardiography findings in infants of diabetic mothers

**DOI:** 10.1186/s13052-025-02178-2

**Published:** 2026-01-23

**Authors:** Sema Nur Taşkın, Vehbi Doğan, Zeynep Kasap Acungil, Rüveyda Gümüşer Cinni, Serap Bilge, Şeyma Ünüvar Gök, Leyla Aydoğan, İsmail Benli, Arzu Er Kara, Deniz Anuk İnce, Resul Yılmaz

**Affiliations:** 1 Department of Pediatrics, Faculty of Medicine, Tokat Gaziosmanpaşa University, Tokat, Türkiye; 2 Division of Pediatric Cardiology, Department of Pediatrics, Faculty of Medicine, Tokat Gaziosmanpaşa University, Tokat, Türkiye; 3 Department of Physiotherapy and Rehabilitation, Faculty of Health Science, Tokat Gaziosmanpasa University, Tokat, Türkiye; 4 Department of Pharmaceutics Services, Tokat Vocational School of Health Services, Gaziosmanpasa University, Tokat, Türkiye; 5 Department of Cardiology, Tokat State Hospital, Tokat, Türkiye; 6 Division of Neonatology, Department of Pediatrics, Faculty of Medicine, Tokat Gaziosmanpaşa University, Tokat, Türkiye

**Keywords:** Infant of diabetic mothers (IDMs), Tissue doppler imaging (TDI), Cardiac biomarkers, Ischemia-modified albumin (IMA), N-terminal pro-brain natriuretic peptide (NT-proBNP), Troponin T (Trop-T)

## Abstract

**Background:**

Maternal diabetes mellitus (DM) significantly affects fetal cardiac and placental structure and function. This study aimed to assess structural and functional cardiac changes in infants of diabetic mothers (IDMs) using echocardiographic data and cord blood cardiac biomarkers.

**Methods:**

M-mode and pulsed-wave tissue Doppler imaging (PW-TDI) echocardiography along with cord blood cardiac markers were evaluated in 40 IDMs and 40 healthy term newborns. Cardiac biomarkers included troponin T (Trop-T), N-terminal pro-brain natriuretic peptide (NT-proBNP), and ischemia-modified albumin (IMA).

**Results:**

Among the diabetic mothers, 87.5% had gestational diabetes mellitus (GDM) and 12.5% had type 1 diabetes mellitus (T1DM). Interventricular septum (IVS) thickness was significantly greater in the IDM group. Of the IDMs, 32.5% had asymmetric septal hypertrophy. Mid-septal tissue Doppler Imaging (TDI) parameters showed significantly altered velocities and time intervals. NT-proBNP and IMA levels were significantly elevated in the IDMs group, while Trop-T was not statistically different. No correlation was found between TDI parameters and biomarker levels.

**Conclusion:**

Cardiac dysfunction and elevated cord blood biomarkers in IDMs reflect subclinical stress. These findings support the utility of TDI and biochemical markers for early cardiac assessment in IDMs. Larger studies are needed to validate the use of these markers for routine screening and to better understand their pathophysiological significance.

## Introduction

Diabetes mellitus is a chronic metabolic disorder characterized by increased insulin resistance and/or β-cell dysfunction, resulting in disturbances in carbohydrate, protein, and lipid metabolism. GDM, defined as glucose intolerance with onset or first recognition during pregnancy, is associated with a range of adverse maternal and neonatal outcomes [1, 2]. Maternal diabetes—whether gestational or pregestational T1DM—can significantly impact fetal cardiac development and placental circulation. IDMs are particularly vulnerable to cardiovascular abnormalities, with congenital heart defects reported in approximately 3–9% of diabetic pregnancies—up to ten times higher than in non-diabetic pregnancies [3]. Even in the absence of structural anomalies, IDMs may exhibit functional disturbances such as myocardial hypertrophy and diastolic dysfunction. Tissue Doppler imaging is a sensitive echocardiographic modality that allows early detection of subclinical myocardial changes in neonates. In parallel, biochemical markers including Trop-T, NT-proBNP, and IMA have emerged as potential indicators of cardiac stress or injury in neonates. Although not yet routinely used in clinical settings, these biomarkers may have important future roles in the risk stratification of IDMs.

This study aims to assess structural and functional cardiac changes in IDMs by evaluating M-mode and TDI parameters and measuring cord blood levels of Trop-T, NT-proBNP, and IMA. These findings were compared with healthy term neonates to explore their utility in early detection of subclinical cardiac dysfunction.

## Materials & methods

This prospective case-control study was conducted at the Department of Pediatrics, Gaziosmanpaşa University Faculty of Medicine, Tokat, Türkiye. The study was approved by the university’s Clinical Research Ethics Committee, and written informed consent was obtained from the parents of all participants.

### Study population

The study included two groups: IDMs and infants of non-diabetic mothers serving as the control group. Each group consisted of 40 term neonates. Within the IDMs group, 87.5% of mothers were diagnosed with GDM, while 12.5% had pregestational (T1DM). The diagnosis of GDM was based on the criteria of the Second International Conference on GDM, and T1DM was defined according to World Health Organization (WHO) guidelines [4].

Exclusion criteria included prematurity (gestational age < 37 weeks), intrauterine growth restriction, low birth weight, small for gestational age, birth asphyxia, low Apgar scores, poor neonatal adaptation, significant respiratory distress, congenital heart disease (except patent foramen ovale and physiologic ductal patency), dysmorphic features, multiple gestation, or absence of maternal glycemic data.

Comprehensive maternal and neonatal data were recorded, including maternal age, diabetes type, HbA1c levels, treatment modality (e.g., insulin, diet), maternal comorbidities, gestational age, birth weight, neonatal sex, and Apgar scores at 1 and 5 min. As the delivery room was unavailable at the study center during the study period, all neonates were delivered via cesarean section. Maternal glycemic control was evaluated using HbA1c levels, with a mean value of 5.67 ± 1.47%, indicating generally adequate glycemic regulation across the IDMs group.

### Echocardiographic assessment

All neonates underwent echocardiographic examination within the first 48 h of life. Assessments were performed in the supine position using a 7 MHz transducer on the SonoSite M-Turbo ultrasound system (SonoSite Inc., WA, USA), in accordance with the guidelines of the American Society of Echocardiography. The evaluation included two-dimensional (2D), color M-mode, and PW-TDI.

Tissue Doppler imaging measurements were acquired from the basal and mid segments of the IVS using the apical four-chamber view, following the method described by Isaaz et al. The following myocardial velocity and time parameters were measured: early diastolic velocity (E), late diastolic velocity (A), systolic velocity (S), ejection time (ET), isovolumic contraction time (IVCT), and isovolumic relaxation time (IVRT). The myocardial performance index (MPI), also known as the Tei index, was calculated using the formula: MPI = (IVCT + IVRT) / ET.

### Cord blood analysis

Umbilical cord blood was collected immediately after delivery. Samples were centrifuged within 30 min and stored at − 80 °C until biochemical analysis. Cord blood levels of Trop-T, NT-proBNP, and IMA were measured using commercial enzyme-linked immunosorbent assay (ELISA) kits (Elabscience^®^, USA), according to the manufacturer’s instructions.

### Statistical analysis

Data were analyzed using SPSS version 21.0 (IBM Corp., Armonk, NY, USA). Continuous variables were expressed as mean ± standard deviation (SD). The Kolmogorov-Smirnov test was used to assess the normality of data distribution. Independent sample t-tests were used for normally distributed variables, while non-normally distributed data were compared using the Mann–Whitney U test. A p-value of < 0.05 was considered statistically significant.

## Results

### Demographic and clinical characteristics

Both the IDMs and control groups consisted of 40 full-term neonates. There were no statistically significant differences between the groups in terms of gestational age, sex distribution, birth weight, or Apgar scores at the 1st and 5th minutes (Table 1). However, maternal age was significantly higher in the IDMs group compared to controls (32.3 ± X vs. 27.4 ± X years, *p* < 0.05).

Maternal glycemic control was evaluated using maternal HbA1c levels. The mean maternal HbA1c value was 5.67 ± 1.47%, indicating generally adequate glycemic regulation across the IDMs group. Demographic and clinical characteristics of the study population are summarized in Table 1.

**Table 1:** Demographic and clinical characteristics of case and control group

		IDMs group (*n*:40)	Control group (*n*:40)	*p*-value
Gender (%)	*Male*	*55*	*50*	
	*Female*	*45*	*50*	
Gestational age (weeks)		38.39±0.79	38.71±0.80	0.081
Birth weight (grams)		3475±539	3356±430	0.280
Apgar score (1st minute)		8.42±0.71	8.37±0.86	0.779
Apgar score (5th minute)		9.00±0.68	8.92±0.57	0.595

Values are presented as mean ± standard deviation unless otherwise indicated. A p-value < 0.05 was considered statistically significant

### Cord blood cardiac markers

Cord blood levels of Trop-T, NT-proBNP, and IMA were measured in both groups. Although mean Trop-T levels were higher in the IDMs group (119.8 ± 45.6 pg/mL) compared to controls (102.5 ± 42.7 pg/mL), the difference did not reach statistical significance (*p* = 0.084).

In contrast, NT-proBNP and IMA levels were significantly elevated in the IDMs group. The mean NT-proBNP level was 2886.1 ± 4280.7 pg/mL in IDMs, compared to 956.3 ± 450.1 pg/mL in controls (*p* = 0.006). Similarly, the mean IMA concentration was 225.5 ± 92.4 ng/mL in the IDMs group, significantly higher than the 147.1 ± 87.4 ng/mL observed in the control group (*p* < 0.001).

These findings suggest that IMA and NT-proBNP may serve as early markers of subclinical cardiac stress in IDMs. The detailed results are presented in Table 2.

**Table 2:** Measurements of cardiac markers measured in the cord blood of the patients

	IDMs group (*n* = 40)	Control group (*n* = 40)	*p*-value
IMA (ng/ml)	225.52± 92.39	147.05 ± 87.41	0.000*
NT-ProBNP (pg/ml)	2886.05± 4280.66	956.30 ± 450.05	0.006*
Trop-T (pg/ml)	119.80± 45.59	102.55 ± 42.65	0.084

*Values are presented as mean ± standard deviation. A p-value < 0.05 was considered statistically significant. (IMA; Ischemia Modified Albumin, NT-pro-BNP; N-Terminal pro-b type Natriuretic Peptide, Trop-T; Troponin-T)

### Electrocardiography

All neonates underwent standard 12-lead surface electrocardiography within the first 48 h of life. There were no statistically significant differences between the IDMs and control groups in terms of heart rate, PR interval, QRS duration, QT interval, or corrected QT interval (QTc). These findings suggest that overt electrical conduction abnormalities are not present in the early neonatal period among infants of diabetic mothers with generally well-controlled glycemia (*p* > 0.05 for all comparisons).

### Echocardiography

#### M-mode echocardiographic parameters

M-mode echocardiographic measurements revealed significantly increased interventricular septal thickness in IDMs compared to the control group. The mean diastolic IVS thickness (IVSd) was 5.17 ± 1.24 mm in the IDMs group versus 4.21 ± 0.76 mm in controls (*p* = 0.001). Similarly, systolic IVS thickness (IVSs) was significantly higher in IDMs (7.07 ± 1.69 mm) than in controls (5.65 ± 1.44 mm, *p* < 0.001). Asymmetric septal hypertrophy, defined as a disproportionate thickening of the IVS, was detected in 13 of 40 IDMs (32.5%). Despite these structural changes, no neonate exhibited clinical signs of hypertrophic cardiomyopathy during the study period. In addition to septal parameters, left ventricular posterior wall thickness during diastole (LVPWd) was significantly lower in the IDMs group (*p* = 0.027), while other dimensions such as LV end-diastolic diameter (LVEDd) and end-systolic diameter (LVESD) were similar between groups. Detailed M-mode echocardiographic findings are summarized in Table 3.

**Table 3:** M-Mode echocardiography data of IDMs and control group

	IDMs group (*n* = 30)	Control group (*n* = 26)	*p*-value
IVSd (mm)	5.17 ± 1.24	4.21 ± 0.76	0.001*
IVSs (mm)	7.07 ± 1.69	5.65 ± 1.44	0.000*
*LVPWd*: (mm)	3.49 ± 0.74	3.85 ± 0.67	0.027*
*LVPWs* (mm)	5.13 ± 1.13	5.54 ± 1.05	0.080
*LVEDd*: (mm)	16.59 ± 2.33	15.56 ± 2.07	0.086
*LVESD* (mm)	9.78 ± 2.36	9.14 ± 1.76	0.197

Values are expressed as mean ± standard deviation. A p-value < 0.05 was considered statistically significant. IVSd: Diastolic Interventricular Septum Thickness, IVSs: Systolic İnterventricular Septal Thickness, LVPWd: Diastolic Left Ventricular Posterior Wall Measurement, LVPWs: Systolic Left Ventricular Posterior Wall Measurement, LVEDd: Left Ventricular End Diastolic Diameter, LVESD: Left Ventricular End-Systolic Diameter

#### Tissue doppler echocardiographic findings

Tissue Doppler imaging was performed on both the basal and mid segments of the IVS to assess myocardial performance in neonates. Significant alterations in both velocity and time interval parameters were observed in IDMs compared to the control group.

In the basal septum, S was significantly lower in the IDMs group (3.99 ± 0.71 cm/s vs. 4.98 ± 1.24 cm/s, *p* = 0.007), and the ET was significantly prolonged (192.5 ± 18.4 ms vs. 165.0 ± 20.3 ms, *p* < 0.001). The IVRT and IVCT were both significantly shorter in IDMs. As a result, MPI, a composite indicator of global ventricular function, was significantly reduced in the IDMs group (*p* < 0.001).

Similarly, measurements from the mid-septum revealed significantly lower E, A, and S wave velocities in the IDMs group, along with prolonged ET and reduced IVRT and IVCT. The MPI from the mid-septum was also markedly lower in IDMs (0.55 ± 0.08 vs. 0.82 ± 0.15, *p* < 0.001), indicating impaired coordination of systolic and diastolic function. These findings are summarized in Table 4.

Collectively, these results reflect subclinical myocardial dysfunction in neonates born to diabetic mothers, even in the absence of overt structural cardiac abnormalities.

**Table 4:** Pulsed-wave tissue Doppler imaging measurements from the IVS

		IDMs group (*n* = 20)	Control Group (*n* = 26)	*p*-value
IVS (basal)	E (cm/s)	5.05 ± 0.97	5.74 ± 1.7	0.081
	A (cm/s)	4.82 ± 0.79	6.19 ± 2.33	0.086
	E/A	1.06 ± 0.20	0.96 ± 0.21	0.180
	S (cm/s)	3.99 ± 0.71	4.98 ± 1.24	0.007*
	ET (ms)	192.5 ± 18.37	165 ± 20.34	0.000*
	IVRT (ms)	48.92 ± 7.64	60.38 ± 10.76	0.001*
	IVCT (ms)	48.21 ± 5.4	63.15 ± 10.76	0.001*
	MPI	0.50 ± 0.08	0.76 ± 0.15	0.000*
IVS (mid)	E (cm/s)	4.09 ± 1.08	4.85 ± 0.96	0.025*
	A (cm/s)	3.97 ± 1.36	5.57 ± 1.68	0.006*
	E/A	1.08 ± 0.22	0.92 ± 0.23	0.021*
	S (cm/s)	2.7 ± 0.84	4.32 ± 1.6	0.002*
	ET (ms)	189.61 ± 14.35	163.72 ± 19.05	0.000*
	IVRT (ms)	51.15 ± 4.16	66.38 ± 13.54	0.000*
	IVCT (ms)	51.92 ± 9.02	67.88 ± 15.75	0.000*
	MPI	0.55 ± 0.08	0.82 ± 0.15	0.000*

Values are presented as mean ± standard deviation. A p-value < 0.05 was considered statistically significant. ET: Ejection Time, IVRT: Isovolumic Relaxation Time, IVCT: Isovolumic Contraction Time, MPI: Myocardial Performance Index

## Discussion

According to the 2023 *International Diabetes Federation* (IDF) *Diabetes Atlas*, the standardized global prevalence of GDM is estimated at 14.0% when applying a universal oral glucose tolerance test (OGTT) strategy in conjunction with the diagnostic criteria proposed by the *International Association of Diabetes in Pregnancy Study Group* (IADPSG) [5]. In a large retrospective cohort study conducted by Chen et al., which included 16,974 pregnant women hospitalized between 2018 and 2019, 2,860 cases of diabetes in pregnancy (DIP) were identified, corresponding to an overall incidence of 17.1%. Of these, 14.85% were classified as GDM and 2% as pregestational DM [6]. In our cohort, 87.5% of diabetic mothers had GDM, a proportion closely aligned with the approximately 86% reported by Chen et al., further supporting the predominance of GDM among DIP cases.

In a prospective observational study by Schierz et al. (2017), transitional cardiovascular and cerebrovascular dynamics in IDMs were comprehensively evaluated using targeted neonatal echocardiography, electrocardiography, and Doppler flow assessments. Despite improvements in maternal glycemic control and the inclusion of largely asymptomatic neonates, the authors reported persistent alterations in cardiac structure and function during the early postnatal period [7]. Among the most frequently observed anomalies in IDMs are cardiovascular malformations, which occur in approximately 3–9% of diabetic pregnancies—up to tenfold more often than in non-diabetic pregnancies [3]. 

Maternal diabetes influences fetal cardiac development throughout gestation. In early pregnancy, it may disrupt gene expression and cardiac morphogenesis, leading to structural anomalies. In later stages, maternal hyperglycemia can result in fetal hyperinsulinemia, stimulating insulin receptors in myocardial cells. Insulin, an anabolic hormone, promotes cellular hypertrophy and hyperplasia, potentially resulting in myocardial thickening. Infants of mothers with pregestational diabetes are at a higher risk for both morbidity and mortality compared to those exposed to GDM [8, 9]. Factors such as suboptimal antenatal care, poor glycemic control in the first trimester, and maternal smoking further elevate the risk of congenital heart disease. Conversely, studies in diabetic animal models have shown that early antenatal administration of antioxidants (e.g., lipoic acid, vitamin C, N-acetylcysteine) may mitigate this risk [3, 10, 11].

Recent studies indicate that transient hyperglycemic peaks during the third trimester—commonly observed in gestational diabetes—play a pivotal role in the development of fetal myocardial hypertrophy and impaired diastolic function [12–14]. Hypertrophic cardiomyopathy is a well-defined cardiac alteration in IDMs, with an incidence of approximately 40% as reported by Al-Biltagi [3]. In the prospective study by Schierz et al., IVS hypertrophy was identified in about 30% of IDMs, particularly among those born to mothers with pregestational diabetes. This hypertrophy showed a significant positive correlation with third-trimester maternal HbA1c levels and cord blood C-peptide concentrations [7]. Similarly, asymmetric septal hypertrophy was observed in 13 infants (32.5%) in the current study, although none exhibited clinical symptoms. These findings align with those reported by Çimen et al. (36%) and Vela-Huerta et al. (50%) [15, 16].

In the study by Darwish et al., the incidence of hypertrophic cardiomyopathy—assessed via fetal M-mode echocardiography at 23–24 and 27–28 weeks of gestation in pregnancies complicated by pregestational type 2 diabetes—was significantly higher in the diabetic group at both the first (33.4%) and second (56.7%) evaluations [17]. Similarly, Sikdar et al. reported that infants born to mothers with gestational diabetes exhibited significantly greater left ventricular posterior wall thickness during diastole compared with those born to non-diabetic mothers [18]. Consistent with these findings, our study identified increased left ventricular posterior wall thickness in 20 infants (66.7%). Furthermore, IVS thickness was significantly higher in the IDMs group, with asymmetric septal hypertrophy present in 13 infants (32.5%), although none exhibited clinical symptoms. A systematic review and meta-analysis by Skovsgaard et al. further demonstrated that maternal diabetes during pregnancy adversely affects myocardial thickness and overall cardiac function in neonates during the first six months of life. While systolic function is generally preserved, diastolic and global cardiac function parameters are often impaired; notably, these alterations tend to regress over time, with no significant differences observed in older children [19]. Additionally, Bogo et al. found that adequate maternal metabolic control during pregnancy may reduce the incidence of hypertrophic cardiomyopathy at birth [12]. Whereas Pooransari et al. reported that fetal cardiac dysfunction may still occur despite optimal glycemic control [20].

Previous research has also described diastolic dysfunction in IDMs, with cardiac hypertrophy considered a major contributor to impaired ventricular relaxation [15, 21]. In our study, we specifically focused on the IVS for PW-TDI due to its high susceptibility to metabolic alterations in IDMs. Doppler analysis revealed lower E, A, and S velocities, accompanied by reduced IVRT, IVCT, and MPI values in the IDMs group compared with controls. This pattern—particularly the concurrent reduction in both E and A wave velocities—suggests a more pronounced impairment in diastolic filling than that reported in some previous studies. For example, Schierz et al. examined mostly asymptomatic neonates and cases with adequate maternal glycemic control, reporting significantly higher mitral and tricuspid E and A wave velocities in the IDMs group compared to controls, thereby supporting the presence of diastolic dysfunction [7]. While they observed reduced ejection fraction (EF) and shortening fraction (FS) at 72 h of life, no significant differences were found in global systo-diastolic parameters such as the MPI. These discrepancies from our results may be attributable to differences in study populations, timing of postnatal assessment, or methodological approaches. Darwish et al. demonstrated that fetuses of diabetic mothers are at increased risk of developing functional cardiac abnormalities even in the absence of structural anomalies, particularly during the third trimester [17]. Their case-control study revealed significant increases in interventricular septal thickness, IVCT, IVRT, aortic acceleration time (AAT), and MPI in fetuses of mothers with pregestational type-2 DM compared to controls, with these alterations progressing over time. Notably, the incidence of hypertrophic cardiomyopathy was markedly higher in the diabetic group at both assessment points. Similarly, Sobeih et al. (2020) evaluated diastolic function in term neonates born to diabetic mothers using both conventional pulsed-wave Doppler and TDI, reporting significantly lower mitral and tricuspid E wave velocities and E/A ratios, as well as reduced septal, left, and right ventricular E′/A′ ratios in IDMs [22]. In contrast, A′ wave velocities were elevated, with abnormalities more pronounced in neonates of mothers with poor glycemic control. Importantly, TDI proved more sensitive than conventional Doppler in detecting left ventricular diastolic dysfunction, which aligns with our observation of a globally impaired diastolic profile. Furthermore, Fatima et al. reported that infants of diabetic mothers exhibited both systolic and diastolic dysfunction, characterized by lower EF, reduced global longitudinal strain, decreased mitral and tricuspid E wave velocities, and reduced E/A ratios, along with increased A wave velocity and elevated E/E′ ratio [23]. They concluded that both poor glycemic control and pregestational diabetes were associated with more severe cardiac dysfunction in neonates. We deliberately selected the basal and mid-septal regions for TDI based on both pathophysiological relevance and technical feasibility in the early neonatal period. The IVS is particularly susceptible to insulin-mediated hypertrophy in IDMs, making it a sensitive region for detecting subclinical myocardial dysfunction. Moreover, septal imaging in the apical four-chamber view provides a stable and reproducible acoustic window in neonates, especially within the first 48 h of life. In contrast, lateral wall measurements are often limited by lung aeration, thoracic compliance, and the more oblique orientation of the lateral myocardial wall. Our approach is in line with previous neonatal echocardiographic studies aiming to capture early cardiac dysfunction in this specific population.

Physiologically, MPI tends to rise shortly after birth and stabilizes after 24 h [12]. In the study by Çimen et al., MPI was higher in IDMs when measured at the left and right ventricular walls, although no significant difference was found in the septal MPI [15]. In contrast, our study identified significantly lower MPI in the IVS of IDMs. The MPI is derived from the sum of IVRT and IVCT divided by ET. In our cohort, while IVRT and IVCT were lower, ET was longer in IDMs. Since left ventricular ejection time is influenced by preload, afterload, geometry, and contractility—and is positively associated with stroke volume—its prolongation in IDMs may reflect compensatory mechanisms to preserve systolic output in the context of hypertrophic remodeling [24]. Notably, the earlier study by Çimen et al. did not report individual values for IVRT, IVCT, or ET, and their subgrouping by presence of hypertrophy may account for the differing results [15]. Sobeih et al. found that IDMs had significantly lower mitral/tricuspid E wave velocities and E/A ratios compared to controls, with these abnormalities being more pronounced in those with poor glycemic control. Additionally, the MPI values measured by tissue Doppler imaging were significantly higher in IDMs [22]. Consistent with these findings, previous studies have also reported a prolonged early diastolic filling deceleration time (E-DT) in IDMs, together with impaired mitral E/A and tissue Doppler–derived E′/A′ ratios. These alterations primarily indicate early diastolic dysfunction due to impaired myocardial relaxation rather than reduced ventricular compliance [22, 25].

While structural anomalies can occur, several studies emphasize that diastolic abnormalities and arrhythmias may also be present in structurally normal hearts of IDMs [15]. In our study, no significant electrocardiographic differences were detected between groups within the first 48 h of life. A recent case–control study (2023–2024) investigated 14 neonatal cases of atrial flutter diagnosed on the first day of life; only one infant was born to a diabetic mother and presented with both an atrial septal defect and ventricular hypertrophy [26]. In another study, Salehi et al. (2023) included 150 neonates—75 born to mothers with well-controlled diabetes and 75 to healthy mothers. Electrocardiographic and echocardiographic evaluations revealed significantly higher QT and QTc intervals in the IDMs group compared with controls [27]. Similarly, Schierz et al. reported that electrocardiographic abnormalities, such as negative T-waves and wandering atrial pacemaker, were significantly more frequent in IDMs [7]. Although no arrhythmias were observed in our cohort, continuous Holter monitoring could be valuable for detecting transient or asymptomatic events.

Cord blood biomarkers are increasingly recognized as early indicators of fetal cardiac stress. In particular, elevated levels of cardiac troponin T (cTnT) and NT-proBNP have been reported in infants born to mothers with gestational or type 1 diabetes compared to healthy controls. In a prospective study conducted by Mert et al. (2016), including 88 neonates born to mothers with either pregestational or gestational diabetes, both NT-proBNP and cTnT concentrations in cord blood were found to be significantly higher than those in the control group. Furthermore, NT-proBNP levels were positively correlated with IVS thickness, supporting its potential role as a biomarker of myocardial strain in this population [28]. Similarly, Lee-Tannock et al. reported that BNP z-scores were significantly associated with both maternal diabetes and obesity, suggesting a potential link between the intrauterine metabolic environment and fetal cardiac stress. Although troponin I (TnI) levels were higher in women with gestational diabetes compared with normoglycemic pregnancies, the difference was not statistically significant. Notably, these biomarker elevations were positively correlated with increased IVS thickness in the diabetic group, supporting the hypothesis that maternal metabolic dysregulation may contribute to subclinical structural cardiac changes in the fetus [29]. In the study conducted by Lehtoranta et al., which included 33 women with type 1 diabetes and 67 controls, umbilical cord serum concentrations of natriuretic peptides and troponin T were found to be significantly higher in the diabetic group compared to the control group [30].

Building on these findings, in the prospective study conducted by Kłaczewska and Tomasiuk (2022), high-sensitivity cardiac troponin T (hs-cTnT) levels were compared between 34 neonates born to mothers with type 1 or type 2 diabetes and 52 healthy controls. The median hs-cTnT level was 0.171 ng/mL in the GDM group and 0.12 ng/mL in the healthy group. Although the GDM group showed a wider interquartile range and a trend toward higher troponin concentrations, the difference was not statistically significant. The authors suggested that hs-cTnT may have potential as a biomarker for subclinical cardiac alterations in infants of diabetic mothers, but emphasized the need for larger, follow-up studies to confirm these findings [31]. In our cohort, Trop-T levels were higher in IDMs but did not reach statistical significance. BNP and its inactive counterpart NT-proBNP are released in response to myocardial wall stress and serve not only as sensitive biomarkers of subclinical cardiac dysfunction, but also act as endogenous cardioprotective agents by modulating volume overload, inhibiting myocardial fibrosis, and attenuating hypertrophic signaling [32–34]. In our study, the diabetic pregnancies were categorized as class A according to the Modified White classification and exhibited relatively well-controlled glycemia (mean HbA1c: 5.67 ± 1.47%); nevertheless, cord blood NT-proBNP levels were significantly higher in the IDMs group compared to healthy controls (*p* = 0.006). This finding supports the notion that even in the absence of overt hyperglycemia, subtle hemodynamic or myocardial alterations may be ongoing in utero, reinforcing the potential utility of NT-proBNP as a screening biomarker for early cardiac dysfunction in IDMs.

Ischemia-modified albumin is a derivative of the N-terminal region of human serum albumin, structurally altered by ischemia-induced acidosis, oxidative damage, membrane disruption, and hypoxia. Unlike conventional cardiac biomarkers such as creatine kinase – myocardial band (CK-MB), TnI, and myoglobin, IMA rises in the serum during the early stages of ischemia, even before irreversible necrosis occurs, rendering it a potentially valuable early marker of tissue injury [35]. Studies specifically evaluating IMA levels in infants of diabetic mothers are extremely limited, highlighting a significant gap in the current research literature. In our study, IMA concentrations measured in venous cord blood collected immediately after cesarean delivery were significantly higher in the IDMs group than in the healthy control group (*p* < 0.001). It should be noted that IMA values in neonates may be influenced by baseline serum albumin levels, as there is a known inverse correlation. Therefore, in cases of hypoalbuminemia, interpretation of IMA should be approached with caution to avoid overestimation of ischemic or oxidative stress. This finding is consistent with existing knowledge that oxidative stress increases and antioxidant enzyme activity decreases in the setting of gestational diabetes mellitus [35].

Despite extensive literature on structural and functional cardiac changes in IDMs, to our knowledge, no prior studies have specifically examined the association between asymmetric septal hypertrophy and IMA levels in this population. In one related investigation, Topaloğlu et al. analyzed venous cord blood from 29 IDMs and 20 term neonates delivered vaginally at 37–41 weeks of gestation. They reported significantly elevated levels of both IMA and mean platelet volume (MPV) in IDMs compared to controls [36]. Similarly, Mohsen et al. demonstrated that both MPV and IMA were significantly elevated in IDMs and were associated with adverse postnatal outcomes, suggesting their potential role as early indicators of oxidative stress-mediated complications [37].

Beyond the neonatal context, elevated IMA levels have also been reported in various adult patient populations, including those with diabetes mellitus, hypertrophic cardiomyopathy, acute ischemic stroke, intracerebral hemorrhage, subarachnoid hemorrhage, and peripheral artery disease [38–40]. IMA, a marker of oxidative stress and ischemia, has been increasingly investigated in the context of type-2 DM due to its potential association with poor glycemic control and vascular complications. In a cross-sectional study by Chawla et al., 82 patients with type-2 DM were evaluated for IMA levels in relation to glycemic control, lipid profile, and diabetic complications. The study demonstrated that IMA levels were significantly higher in patients with poor glycemic control (HbA1c >7.0%) compared to those with well-controlled diabetes (*p* < 0.05), and a moderate positive correlation was identified between IMA and HbA1c levels (r² = 0.14). Although not statistically significant, IMA levels also tended to increase in patients with lower HDL cholesterol and in those who had developed complications such as neuropathy and retinopathy. These findings suggest that IMA may reflect underlying subclinical ischemia associated with chronic hyperglycemia and support its utility as a biochemical marker of long-term oxidative stress in type-2 DM [39]. Recent evidence from a 2024 systematic review and meta-analysis (Zinellu et al., 2024) indicates that elevated IMA levels are significantly associated with type 1 and type 2 diabetes, even in pre-diabetic conditions, underscoring its potential as a sensitive biomarker of oxidative stress and endothelial injury [41]. In a study investigating neonates with congenital heart disease, IMA levels were found to be significantly higher in the postoperative period compared to healthy controls, suggesting that IMA may reflect ongoing ischemic burden and could have prognostic value in critically ill newborns [42].

Currently, the clinical use of biomarkers such as NT-proBNP and IMA in cord blood is primarily investigational. However, our findings and similar studies suggest that these markers may help detect early subclinical cardiac stress in neonates. With further validation in large-scale cohorts, they could potentially be incorporated into routine neonatal assessments.

In our cohort, no statistically significant correlation was identified between the cardiac markers and echocardiographic parameters, including interventricular septal thickness and tissue Doppler velocities. This finding implies that while IMA reflects systemic oxidative stress, it may not directly mirror localized structural or functional myocardial alterations detectable by echocardiography in the early neonatal period.

### Limitation

This study has several limitations that should be acknowledged. First, all neonates in both the case and control groups were delivered via cesarean section. This was not a study design preference but rather a consequence of logistical constraints, as no active delivery room was available at the time of data collection. Although this uniform mode of delivery eliminates variability between groups and allows for more controlled comparisons, it may limit the generalizability of our findings to vaginally delivered neonates. Additionally, the sample size, while statistically adequate for the observed differences, may not capture the full spectrum of subclinical myocardial dysfunction, especially in relation to biochemical markers. Furthermore, although significant differences were found between groups in both cardiac biomarkers and echocardiographic parameters, no statistically significant correlations were identified between these variables within the IDMs group. This may be attributed to the limited sample size or the complex, non-linear interaction between structural and biochemical cardiac changes in utero. Lastly, this study represents a single-center experience and reflects the specific demographic and clinical characteristics of the population served, which may not be representative of broader populations.

## Conclusion

Gestational diabetes mellitus has a profound impact on fetal cardiac structure and function, with potential consequences extending into the postnatal period and even adulthood. Effective prevention, early diagnosis, and optimal glycemic control during pregnancy are critical strategies to mitigate these risks.

In this study, we observed elevated levels of Troponin-T, NT-proBNP, and IMA in the cord blood of IDMs. These elevations are likely reflective of subclinical cardiac hypertrophy, diastolic dysfunction, and heightened oxidative stress in utero. Although no significant correlations were found between cardiac biomarker levels and echocardiographic parameters such as septal thickness or TDI indices, the presence of these biochemical alterations underscores the complexity of fetal cardiac adaptation in diabetic pregnancies. This lack of correlation may reflect the limitations of our sample size or the complex, asynchronous progression of structural, functional, and biochemical cardiac changes in utero. We conclude that further multicenter, longitudinal studies with larger sample sizes and postnatal follow-up are warranted to elucidate the prognostic value of these markers and to establish early screening strategies for infants at increased cardiovascular risk. 

## Data Availability

The datasets used and/or analyzed during the current study are available from the corresponding author upon reasonable request.

## References

[1] Ye W, Luo C, Huang J, Li C, Liu Z, Liu F. Gestational diabetes mellitus and adverse pregnancy outcomes: systematic review and meta-analysis. BMJ. 2022;377:e067946. 35613728 10.1136/bmj-2021-067946 PMC9131781

[2] Harreiter J, Roden M. Diabetes mellitus-Definition, classification, diagnosis, screening and prevention (Update 2019)]. Wien Klin Wochenschr. 2019;131:6–15. 30980151 10.1007/s00508-019-1450-4

[3] Al-Biltagi M, El Razaky O, El Amrousy D. Cardiac changes in infants of diabetic mothers. World J Diabetes. 2021;12(8):1233–47. 34512889 10.4239/wjd.v12.i8.1233 PMC8394229

[4] Diagnostic criteria and classification of hyperglycaemia first detected in pregnancy: a world health organisation guideline. Diab Res Clin Pract. 103(3):341–63. 10.1016/j.diabres.2013.10.012 24847517

[5] Wang H, Li N, Chivese T, Werfalli M, Sun H, Yuen L, Hoegfeldt CA, Powe CE, Immanuel J, Karuranga S, Divakar H, Levitt N, Li C, Simmons D, Yang X, IDF Diabetes Atlas Committee, Hyperglycaemia in Pregnancy Special Interest Group IDF diabetes atlas: Estimation of global and regional gestational diabetes mellitus prevalence for 2021 by international association of diabetes in pregnancy study group’s criteria. Diabetes Res Clin Pract. 2022;183:109050. . 10.1016/j.diabres.2021.109050. 34883186 10.1016/j.diabres.2021.109050

[6] Chen J, Wang Z, Wu W, Chen H, Zhong C, Liang L, Li Y Clinical analysis of 2860 cases of diabetes in pregnancy: a single-center retrospective study. BMC Pregnancy Childbirth. 2022;22(1):418. . 10.1186/s12884-022-04712-. 35585514 10.1186/s12884-022-04712-0 PMC9118638

[7] Schierz IAM, Pinello G, Piro E, Giuffrè M, La Placa S, Corsello G Transitional hemodynamics in infants of diabetic mothers by targeted neonatal echocardiography, electrocardiography and peripheral flow study. The journal of maternal-fetal & neonatal medicine: the official journal of the European association of perinatal Medicine, the federation of Asia and Oceania perinatal societies. Int Soc Perinat Obstetricians. 2018;31(12):1578–85. . 10.1080/14767058.2017.1320544. 10.1080/14767058.2017.1320544 28412852

[8] Blonde L, Umpierrez GE, Reddy SS, et al. American association of clinical endocrinology clinical practice guideline: developing a diabetes mellitus comprehensive care plan-. Endocr Pract. 2022;28(10):923–1049. 35963508 10.1016/j.eprac.2022.08.002 PMC10200071

[9] Kautzky-Willer A, Winhofer Y, Kiss H, et al. Gestations diabetes (GDM) (Update 2023). Wien Klin Wochenschr. 2023;135:115–28. 37101032 10.1007/s00508-023-02181-9 PMC10132924

[10] Depla AL, De Wit L, Steenhuis TJ, et al. Effect of maternal diabetes on fetal heart function on echocardiography: systematic review and meta-analysis. Ultrasound Obstet Gynecol. 2021;57(4):539–50. 32730637 10.1002/uog.22163 PMC8048940

[11] Zhao Z. Reevaluation of antioxidative strategies for birth defect prevention in diabetic pregnancies. J Biomol Res Ther. 2016;5(3):145. 28824831 10.4172/2167-7956.1000145 PMC5560165

[12] Bogo MA, Pabis JS, Bonchoski AB, et al. Cardiomyopathy and cardiac function in fetuses and newborns of diabetic mothers. J Pediatr (Rio J). 2021;97(5):520–4. 33176166 10.1016/j.jped.2020.10.003 PMC9432014

[13] Diniz MS, Hiden U, Falcão–Pires I, Oliveira PJ, Sobrevia L, Pereira SP. Fetoplacental endothelial dysfunction in gestational diabetes mellitus and maternal obesity: a potential threat for programming cardiovascular disease. Biochim Et Biophys Acta – Mol Basis Disease. 2023;1869(8). Article 166834. 10.1016/j.bbadis.2023.166834. 10.1016/j.bbadis.2023.166834 37541330

[14] Ghandi Y, Habibi D, Nasri K, Alinejad S, Taherahmad H, Shabestari A, Nematinejad A. Effect of well-controlled gestational diabetes on left ventricular diastolic dysfunction in neonates. J Maternal-Fetal Neonatal Med. 2019;32(13):2101–6. . 10.1080/14767058.2018.1425832. 10.1080/14767058.2018.1425832 29911451

[15] Çimen D, Karaaslan S. Evaluation of cardiac functions of infants of diabetic mothers using tissue doppler echocardiography. Türk Ped Arş. 2014;49:25–9. 10.5152/tpa.2014.843 PMC4462271 26078628

[16] Vela-Huerta MM, Amador-Licona N, Orozco Villagómez HV. Asymmetric septal hypertrophy in appropriate for gestational age infants born to diabetic mothers. Indian Pediatr. 2019;56(4):314–6. 10.1007/s13312-019-1522-6. 31064902

[17] Darwish A, Abdel-Raouf M, Kamel R, Salah E, Salah M, Okasha A. Fetal echocardiographic parameters in pregnancies complicated by diabetes: a case control study. BMC Pregnancy Childbirth. 2022;22(1):650. 10.1186/s12884-022-04969-5. 35982412 10.1186/s12884-022-04969-5 PMC9386926

[18] Sikdar J, Das PS, Halder D, Banerjee J, Mukhopadhyay B, Rana A, Karan S. Echocardiographic evaluation of infants born to mothers with gestational diabetes mellitus: A cross-sectional analytical study. J Clin Diagn Res. 2024;18(7):CC01–5. 10.7860/JCDR/2024/71042.19659.

[19] Skovsgaard CB, Møller A, Bjerre JV, Kampmann U, Kyng KJ. Diabetes in pregnancy and offspring cardiac function: A systematic review and meta-analysis. Front Pead. 2024;12:1404625. 10.3389/fped.2024.1404625. 10.3389/fped.2024.1404625 PMC11291373 39091986

[20] Pooransari P, Mehrabi S, Mirzamoradi M, Salehgargari S, Afrakhteh M. Comparison of parameters of fetal doppler echocardiography between mothers with and without diabetes. Int J Endocrinol Metabolism. 2022;20(4):e117524. 10.5812/ijem-117524. 10.5812/ijem-117524 PMC9884331 36741331

[21] Xie W, Wang Y, Xiao S, Qiu L, Yu Y, Zhang Z. Association of gestational diabetes mellitus with overall and type specific cardiovascular and cerebrovascular diseases: systematic review and meta-analysis. BMJ. 2022;378:e070244. Published 2022 Sep 21. 36130740 10.1136/bmj-2022-070244 PMC9490552

[22] Sobeih AA, Sakr MMA, Abolmaaty RK. Assessment of cardiac diastolic function in infants of diabetic mothers using tissue doppler echocardiography. Egypt Pediatr Association Gaz. 2020;68(1):10.

[23] Fatima M, Abqari S, Ali SM, Hakim S. Echocardiographic evaluation of cardiac function in infants of mothers with diabetes. J Indian Acad Echocardiography Cardiovasc Imaging. 2023;7(2):85–92. 10.4103/jiae.jiae_26_23.

[24] Alhakak AS, Teerlink JR, Lindenfeld J, Böhm M, Rosano GMC, Biering-Sørensen T. The significance of left ventricular ejection time in heart failure with reduced ejection fraction. Eur J Heart Fail. 2021;23(4):541–51. 10.1002/ejhf.2125. 33590579 10.1002/ejhf.2125

[25] Ghandi Y, Habibi D, Nasri K, Alinejad S, Taherahmad H, Arjmand Shabestari A, Nematinejad A. Effect of well-controlled gestational diabetes on left ventricular diastolic dysfunction in neonates. The journal of maternal-fetal & neonatal medicine: the official journal of the European association of perinatal Medicine, the federation of Asia and Oceania perinatal societies. Int Soc Perinat Obstetricians. 2019;32(13):2101–6. 10.1080/14767058.2018.1425832. 10.1080/14767058.2018.1425832 29911451

[26] Park SH, Lim G, Oh KW, Ko JK. Neonatal atrial flutter: clinical characteristics of 14 cases in a single center. Neonatal Med. 2022;29(3):97–103. 10.5385/nm.2022.29.3.97.

[27] Salehi F, Boghrati M, Fanoodi A, Ghoncheh M, Rezaei M, Salehiniya H, Hajipoor H. Electrocardiography and echocardiography findings in infants of diabetic mothers: A case–control study. Curr Res Med Sci. 2023;7(1):43–50. 10.22088/crms.7.1.43.

[28] Mert MK, Satar M, Özbarlas N, Yaman A, Özgünen FT, Asker HS, Çekinmez EK, Tetiker T. Troponin T and NT probnp levels in Gestational, type 1 and type 2 diabetic mothers and macrosomic infants. Pediatr Cardiol. 2016;37(1):76–83. 10.1007/s00246-015-1242-1. 26266327 10.1007/s00246-015-1242-1

[29] Lee-Tannock A, Hay K, Kumar S. Differences in biomarkers of cardiac dysfunction in cord blood between normal pregnancies and pregnancies complicated by maternal diabetes. Aust N Z J Obstet Gynaecol. 2022;62(1):79–85. 10.1111/ajo.13415. 34370294 10.1111/ajo.13415

[30] Lehtoranta L, Haapsamo M, Vuolteenaho O, Palo P, Ekholm E, Räsänen J. Fetal cardiovascular hemodynamics in type 1 diabetic pregnancies at near-term gestation. Acta Obstet Gynecol Scand. 2021;100(2):263–71. 10.1111/aogs.13987. 32880890 10.1111/aogs.13987

[31] Kłaczewska J, Tomasiuk R. Cardiac troponin T in neonates from normal and gestational diabetes mellitus pregnancy. BioMed Res Int. 2022:6171687. 10.1155/2022/6171687. 10.1155/2022/6171687 PMC9596251 36303588

[32] Giovou AE, Gladka MM, Christoffels VM. The impact of natriuretic peptides on heart Development, Homeostasis, and disease. Cells. 2024;13(11):931. 10.3390/cells13110931. 38891063 10.3390/cells13110931 PMC11172276

[33] Sarzani R, Allevi M, Di Pentima C, Schiavi P, Spannella F, Giulietti F. Role of cardiac natriuretic peptides in heart structure and function. Int J Mol Sci. 2022;23(22):14415. 10.3390/ijms232214415. 36430893 10.3390/ijms232214415 PMC9697447

[34] Fu S, Ping P, Wang F, Luo L, Sethi G, Ye L. Synthesis, secretion, function, metabolism and application of natriuretic peptides in heart failure. J Biol Eng. 2018;12. Article 2. 10.1186/s13036-017-0093-0. 10.1186/s13036-017-0093-0 PMC5766980 29344085

[35] Shevtsova A, Gordiienko I, Tkachenko V, Ushakova G. Ischemia-modified albumin: origins and clinical implications. Dis Markers. 2021:9945424. 10.1155/2021/9945424. 10.1155/2021/9945424 PMC8315882 34336009

[36] Topaloğlu N, Yıldırım Ş, Tekin M, et al. Mean platelet volume and ischemia modified albumin levels in cord blood of infants of diabetic mothers. Pediatr Neonatol. 2014;55(6):455–8. 24855974 10.1016/j.pedneo.2014.02.002

[37] Mohsen L, Akmal DM, Ghonaim EKE, Riad NM. Role of mean platelet volume and ischemia modified albumin in evaluation of oxidative stress and its association with postnatal complications in infants of diabetic mothers. J Matern Fetal Neonatal Med. 2018;31:1819–23. 28502205 10.1080/14767058.2017.1330329

[38] Dash P, Mangaraj M, Ray S, et al. Ischaemia modified albumin an indicator of widespread endothelial damage in diabetes mellitus. J Physiobiochemical Metabolism. 2014;3:1.

[39] Chawla R, Loomba R, Guru D, Loomba V. Ischemia modified albumin (IMA) - A marker of glycaemic control and vascular complications in type 2 diabetes mellitus. J Clin Diagn Research: JCDR. 2016;10(3):BC13–6. 10.7860/JCDR/2016/15282.7432. 27134857 10.7860/JCDR/2016/15282.7432 PMC4843243

[40] Mangoni AA, Zinellu A. A systematic review and meta-analysis of serum concentrations of Ischaemia-modified albumin in acute ischaemic stroke, intracerebral haemorrhage, and subarachnoid haemorrhage Biomolecules 2022;12(5):653. 10.3390/biom12050653. 35625582 10.3390/biom12050653 PMC9138392

[41] Zinellu A, Mangoni A. A systematic review and meta-analysis of ischemia-modified albumin in diabetes mellitus. Heliyon. 2024;10(16):e35953. 10.1016/j.heliyon.2024.e35953. 39224304 10.1016/j.heliyon.2024.e35953 PMC11366936

[42] Korkut DG, Özlü F, Atlı G, Yıldız ŞM, Demir F, Göçen U, Yapıcıoğlu H. Ischemic-Modified albumin in neonates with congenital heart defects. Am J Perinatol. 2024;41:e827–32. 10.1055/s-0042-1758488. 36584690 10.1055/s-0042-1758488

